# Cencurut virus: A novel *Orthonairovirus* from Asian house shrews (*Suncus murinus*) in Singapore

**DOI:** 10.1016/j.onehlt.2023.100529

**Published:** 2023-03-29

**Authors:** Dolyce H.W. Low, Lena Ch'ng, Yvonne C.F. Su, Martin Linster, Rong Zhang, Yan Zhuang, Mackenzie L. Kwak, Sophie A. Borthwick, Alan T. Hitch, Gavin J.D. Smith, Ian H. Mendenhall

**Affiliations:** a Programme in Emerging Infectious Diseases, Duke-NUS Medical School, 169857, Singapore; b Department of Biological Sciences, National University of Singapore, 16 Science Drive 4, 117558, Singapore; c Museum of Wildlife and Fish Biology, Department of Wildlife, Fish and Conservation Biology, University of California, Davis, CA 95616, USA; d Centre for Outbreak Preparedness, Duke-NUS Medical School, 169857, Singapore; e SingHealth Duke-NUS Global Health Institute, SingHealth Duke-NUS Academic Medical Centre, 168753, Singapore; f Duke Global Health Institute, Duke University, Durham, NC 27710, USA

**Keywords:** Metagenomics, Phylogeny, Thiafora

## Abstract

*Orthonairovirus* is a genus of viruses in the family *Nairoviridae*, order *Bunyavirales,* with a segmented circular RNA genome. They typically infect birds and mammals and are primarily transmitted by ectoparasites such as ticks. Four of nine *Orthonairovirus* genogroups can infect humans, with Crimean-Congo hemorrhagic fever virus infections displaying case fatality rates up to 40%. Here, we discover and describe a novel *Orthonairovirus* as Cencurut virus (CENV). CENV was detected in 34 of 37 Asian house shrews (*Suncus murinus*) sampled in Singapore and in a nymphal *Amblyomma helvolum* tick collected from an infected shrew. Pairwise comparison of CENV S, M, and L segments had 95.0 to 100% nucleotide and 97.5 to 100% amino acid homology within CENV genomes, suggesting a diverse viral population. Phylogenetic analysis of the individual gene segments showed that CENV is related to Erve, Lamgora, Lamusara, and Thiafora viruses, with only 49.0 to 58.2% nucleotide and 41.7 to 61.1% amino acid homology, which has previously been detected in other shrew species from France, Gabon, and Senegal respectively. The high detection frequency suggests that CENV is endemic among *S. murinus* populations in Singapore. The discovery of CENV, from a virus family with known zoonotic potential, underlines the importance of surveillance of synanthropic small mammals that are widely distributed across Southeast Asia.

## 1 Introduction

*Orthonairovirus* is one of the seven genera classified taxonomically in the family *Nairoviridae* within the order *Bunyavirales* comprising nine phylogenetically distinct genogroups, consisting of over 40 virus species [[1], [2], [3], [4]]. Orthonairoviruses are enveloped viruses of approximately 80–120 nm in diameter. The virions consist of three segmented negative-sense RNA designated as small (S), medium (M), and large (L) gene segments, forming circular ribonucleoprotein complexes. These three segments encode for the nucleoprotein (N), glycoproteins (GP, Gn, and Gc subunits), and RNA-dependent RNA polymerase (RdRp), respectively.

Orthonairoviruses are predominantly tick-borne and are distinguished based on their respective dominant vector, soft ticks (family Argasidae) or hard ticks (family Ixodidae) [3,5]. They are distributed across the Old World and infect various vertebrate hosts, including birds, rodents, shrews, ruminants, bats, and humans [3]. Orthonairoviruses have caused disease in humans with varying severity, ranging from asymptomatic and mild to severe hemorrhagic fevers [6]. Crimean-Congo hemorrhagic fever virus (CCHFV) is the most pathogenic *Orthonairovirus* in humans, with high case fatality, and is endemic in Africa and Central Asia [7]. In 2022, over 200 cases were detected in Iraq, with a majority of positive cases reported from individuals with occupational exposure to livestock and a case fatality rate of 16.4% [8]. Other orthonairoviruses that have been reported to cause human diseases include Dugbe virus [9], Issyk-Kul virus [10], Kasokero virus [11], Nairobi sheep disease virus [12], Songling virus [13], Tacheng tick virus [14], and Yezo virus [15], emphasizing the public health burden caused by orthonairoviruses.

Small mammals and bats are significant reservoirs for emerging zoonotic diseases, with potential risks to global health security, including many *Orthonairovirus* species [[16], [17], [18]]. Specifically, small mammals are reservoirs for two *Orthonairovirus* genogroups – Qalyub and Thiafora. The Qalyub genogroup (*Chim* and *Qalyub orthonairoviruses*) are transmitted by soft ticks that infect the order Rodentia (rats and mice) and has been found in Africa and Central Asia [3]. The Thiafora genogroup infects members of the order Soricomorpha (shrews) via an unknown vector [3] and consists of four Orthonairovirus species. Thiafora virus (TFAV) was first discovered in a *Crocidura* shrew species from Senegal in 1971 [18]. Erve virus (ERVEV) was first identified in the greater white-toothed shrews (*Crocidura russulata*) from France in 1982 and has been implicated in severe headache (thunderclap headache) and intracerebral hemorrhage in humans [19,20]. In addition, Lamgora virus (LMGV) and Lamusara virus (LMSV) were recently discovered in 2021 from *Crocidura goliath* and other small mammal species in Gabon [21].

Asian house shrews, *Suncus murinus* (family Soricidae, subfamily Crocidurinae), are widely distributed throughout Southeast and South Asia. Here, we identify and describe a novel *Orthonairovirus* from wild *S. murinus* and a tick from Singapore. We show that the virus is genetically diverse among individual shrews, with high frequency, suggesting it is endemic among *S. murinus* populations in Singapore.

## 2 Material and methods

### 2.1 Shrew and ectoparasite sample collection

**Fig. 1:**
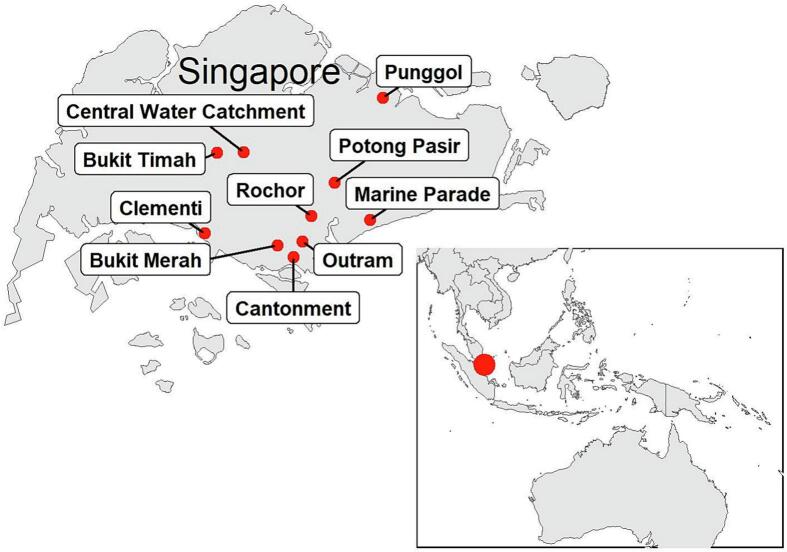
Locations (n = 10) where *Suncus murinus* were trapped in Singapore. Bukit Timah, Cantonment, and Marine Parade sites were categorized into urban and forest, totaling 13 sites.

**Supplementary Fig. 1:**
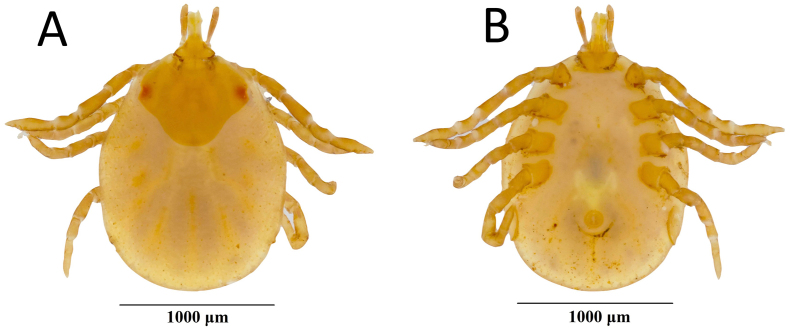
***Amblyomma helvolum*****nymph (A: dorsal view; B: ventral view)**.

Asian house shrews (*Suncus murinus*) (*n* = 37) were trapped using Sherman traps from 13 different locations (urban and forested) across Singapore from April 2012 to February 2014 (Fig. 1). Trapped shrews were euthanized using isoflurane. The carcasses were dissected in a biosafety cabinet in the laboratory, where lung, spleen, and kidney were harvested and stored individually in viral transport media or PBS at −80 °C until processed. Dissection tools were disinfected between each tissue and animal. A single ectoparasite was collected from the snout of a shrew, stored in 80% ethanol, and identified as *Amblyomma helvolum* through taxonomic identification based on local *Amblyomma* tick reports [22] and Southeast Asia reference specimen collection (belonging to author MLK) (Fig. S1). *Amblyomma helvolum* is characterized by having a scutum, festoons, rounded lateral margins of basis *capituli,* and elongated palps.

### 2.2 Sample processing and viral RNA extraction

To perform initial exploratory metagenomic analysis, we selected 20 *S. murinus* and pooled their tissues for RNA extraction and sequencing. Approximately 1–2 g of tissue (lung, spleen, and kidney) was homogenized in 1 ml of AVL buffer (Qiagen, Hilden, Germany) using silicon carbide shards and a Mini-Beadbeater-96 cell disrupter (Biospec Products, Bartlesville, USA) for 1 min at 2000 rpm. The homogenates were pooled and processed following our previous protocol [23], and RNA was extracted using Direct-zol RNA miniprep kit (Zymo Research Corporation, Irvine, USA) as per the manufacturer's instructions. For *S. murinus* (*n* = 37) molecular screening and subsequent sequencing, the lung, spleen, and kidney tissues from each individual shrew were separately weighed (1.3 to 27.6 mg) and homogenized in 500 μl of AVL Buffer (Qiagen, Hilden, Germany). The whole tick was homogenized as above. Homogenates were centrifuged at 13,000 rpm for 1 min, and the RNA extraction was performed on the supernatant as described above.

### 2.3 Metagenomics and virus discovery

Library preparation for metagenomic analysis was prepared directly from the pooled homogenized lung, spleen, and kidney RNA using TruSeq RNA library preparation kit (Illumina, San Diego, USA) following the manufacturer's instructions. The library was quality checked with a Bioanalyzer (Agilent Technologies, Santa Clara, USA) and sequenced on an Illumina MiSeq platform with 150 bp single-end reads. The resulting FASTQ files were quality checked, followed by the removal of low-quality reads (phred score < 20) and index sequences with Cutadapt v2 [24]. Trimmed reads were taxonomically classified using DIAMOND v0.9, and the contigs were used to blast against a local protein database downloaded from NCBI (as of September 2019) [25]. The reads were then assigned to taxonomic classes using MEGAN6 [26].

### 2.4 Novel orthonairovirus quantification

To determine viral copy number in *S. murinus* lung, spleen, and kidney tissues, as well as the *A. helvolum* tick, we performed real-time qPCR using in-house designed primers, probes, and positive control (450 bp minigene plasmid, pUCIDT Amp, Integrated DNA Technologies, Coralville, USA) for the L gene segment from the metagenomic dataset. The qPCR assay was conducted using the AgPath-ID One-Step RT-PCR kit (Applied Biosystems, Waltham, USA) according to the manufacturer's instructions. The 25 μl reaction contained 0.1 μM of each L gene forward (5’-AGCTCTGTAACATCACCAAG-3′) and reverse (5’-CAAGCACTAAAGGACCAAAC-3′) primers, 0.6 μM probe (FAM-AGCAATGTATAACAGCGTCCC-BHQ1), 12.5 μl of 2× buffer, 1 μl of 25× reverse transcriptase enzyme mix and 5 μl of viral RNA template. The PCR cycling conditions were as follows: 1 cycle of 50  °C for 15 mins and 95 °C for 15 mins, 45 cycles of 95 °C for 15 s and 54 °C for 30 s. A standard curve was generated from a triplicate run using a dilution range of positive control plasmid. The optimized qPCR assay had an R^2^ of 0.997, an amplification efficiency of 1.89, a limit of detection (LOD) of 88.63 copies per reaction, and a limit of quantification (LOQ) of 149.37 copies per reaction (34.59 threshold cycle) cut off. Viral RNA copy number was measured in copies per mg of tissue based on the standard curve equation.

### 2.5 Novel orthonairovirus whole genome sequencing

For whole genome sequencing of the novel *Orthonairovirus*, we selected the tick and 27 shrew kidney samples from different sampling occasions and/or sites and with the highest viral RNA copy number. An enriched NGS library was prepared using the Comprehensive Viral Research Panel (Twist Bioscience, San Francisco, USA) coupled with a custom probe set, including oligos designed using the novel *Orthonairovirus* genome from the metagenomic dataset. The Twist EF library prep kit (Twist Bioscience, San Francisco, USA) was used to prepare an Illumina compatible NGS library (see Supplementary methods) that was sequenced as above with 150 bp paired-end reads. De novo assembly was conducted using SPAdes v3 [27], and the resulting contigs were classified by DIAMOND and MEGAN6 as described above.

### 2.6 Phylogenetic analyses

We analyzed full sequence segments from the S (*n* = 28), M (n = 28), and L (*n* = 27) gene segments of the novel *Orthonairovirus,* along with related representative *Orthonairovirus* sequences from GenBank, including Avalon virus (AVAV), Crimean-Congo hemorrhagic fever virus (CCHFV), Dugbe virus (DUGV), Erve virus (ERVEV), Hazara virus (HAZV), Kupe virus (KUPEV), Lamgora virus (LMGV), Lamusara virus (LMSV), Meram virus (no standard abbreviation), Nairobi sheep disease virus (NSDV), Rondonia virus (no standard abbreviation), Thiafora virus (TFAV) and Tofla virus (TLFV). Multiple sequence alignments were performed using transAlign [28] and MAFFT v.7 [29] as implemented in Geneious v11 (Biomatters Ltd., Auckland, New Zealand). We reconstructed individual gene phylogenies based on three datasets (55 S, 54 M, and 57 L gene sequences), using maximum likelihood method in RAxML v8 [30] under the general time reversible (GTR) + GAMMA nucleotide substitution model and branch support was assessed using 1000 bootstrap replicates.

### 2.7 Statistical analysis

A hierarchical Bayesian model was used to determine the probability of *Orthonairovirus* positivity by sex, age, and habitat type. The hierarchical level was “district.” Districts north and northeast were combined for modeling purposes. Priors were chosen to be weakly regularizing to control for both under and overfitting of the model to the data. Convergence criteria, such as effective sample sizes and R-hat values, were used to check for appropriate model convergence throughout, and trace plots were inspected for signs of incomplete mixing when necessary. Non-centered parameterization was used to facilitate convergence and efficient sampling of chains. Compatibility intervals (89%) and contrasts were calculated to evaluate differences among sex, age, and habitat type. R Core Team [31] and the ‘rethinking’ package [32] were used to fit models and generate figures.

## 3 Results

Metagenomic analysis of the pooled tissues from the lung, spleen, and kidney of 20 *Suncus murinus* individuals from Singapore yielded 153,761 reads. Approximately 69.3% of the reads were classified as eukaryotic or bacterial, and 6.9% (*n* = 10,391) were classified as viral. Of these viral reads, 95.4% (*n* = 9914) were classified as *Bunyavirales*, of which 94.2% (*n* = 9338) were assigned as Erve virus (ERVEV). Subsequent phylogenetic analysis identified this as a novel *Orthonairovirus* distinct from ERVEV that we named Cencurut virus (CENV) from the Malay word for ‘shrew.’ Other viral reads detected in the dataset include retroviruses and bacteriophages. NGS datasets are available at the NCBI Sequence Read Archive under BioProject accession number PRJNA777364.

**Fig. 2:**
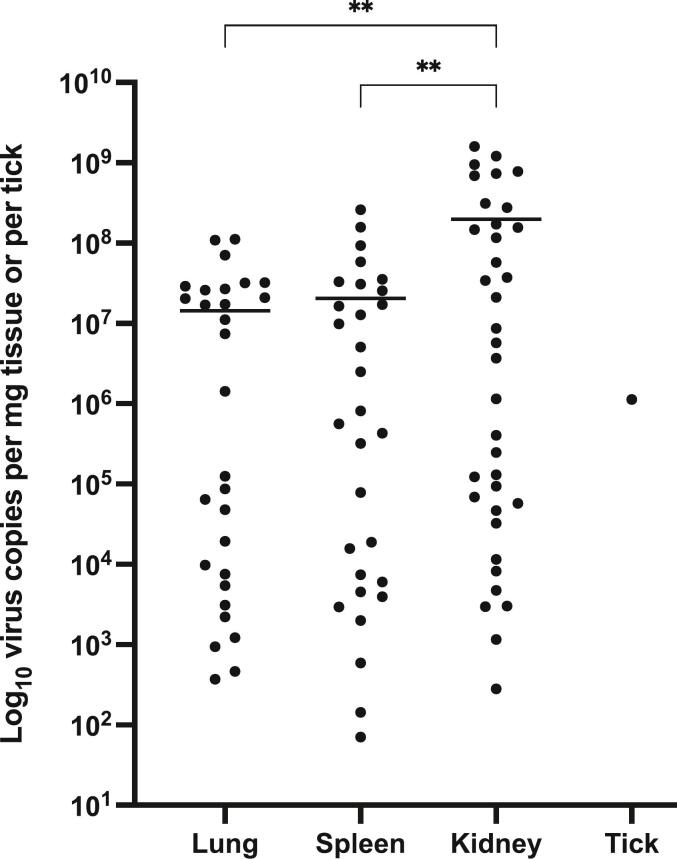
Cencurut virus copies per mg of tissue or tick. Asterisks indicate significance (**P < 0.01) between the mean of each tissue type by one-way ANOVA. *P* = 0.0038 (lung vs. kidney) and *P* = 0.0056 (spleen vs. kidney).

**Supplementary Fig. 2:**
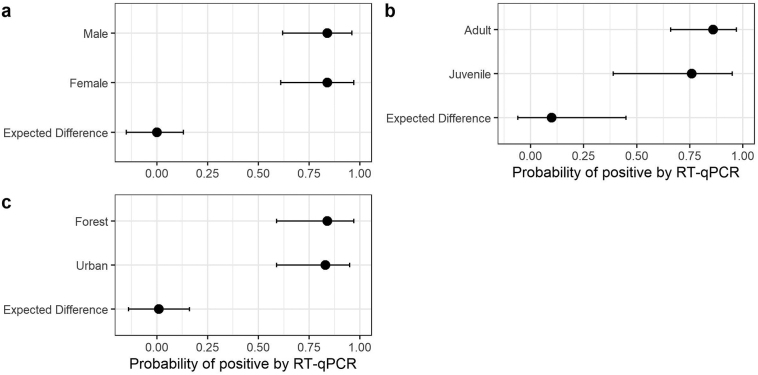
**Hierarchal Bayesian model calculating the probability of a) sex, b) age and c) habitat type affecting the probability of*****Suncus murinus*****being tested positive for Cencurut virus (CENV) by RT-qPCR.**

Thirty-four of 37 *S. murinus* individuals from 12 of 13 sampling sites were RT-qPCR positive for CENV (Table 1, Fig. 2). These positive shrews were trapped from April to December 2012, March to July 2013, and February 2014. Hierarchal Bayesian modeling showed that the probability of a shrew being infected with CENV was not affected by age, sex, or habitat type (Fig. S2). The detection frequency for CENV in lung, spleen, and kidney was 24, 25, and 33 out of 37, respectively. Average viral RNA copy number from shrew lung, spleen, and kidney tissues were calculated at 2.2 × 10^7^, 3.0 × 10^7^, and 2.2 × 10^8^ copies per mg of tissue, respectively (Supplementary Table 1). Kidneys of *S. murinus* displayed the highest viral loads, up to 1.6 × 10^9^ copies per mg of kidney tissue (host ID SM-26). An *Amblyomma helvolum* tick, collected from a CENV-positive shrew (host ID SM-20), was also positive for CENV with 1.1 × 10^6^ viral copies in the entire tick (estimated weight of 1.1 mg).

Whole genome sequencing of CENV from the kidneys of 28 PCR-positive *S. murinus* individuals and the tick generated full-length coding regions of the S (1737 bp, 579aa), M (3591 bp, 1196aa), and L (11,664 bp, 3888aa) CENV gene segments. Interestingly, a 3-nucleotide deletion (positions 1655–1657) was present in the S gene segment of one shrew CENV (host ID SM-15), resulting in a single amino acid deletion while maintaining the reading frame. CENV from two shrews (SM-16 and 17) differed by only a single nucleotide, while the remaining 25 shrews and the tick CENV strains displayed substantial genetic variation (nucleotide identity: 95.0 to 98.6%, nucleotide difference: 237 to 851 nucleotides; amino acid identity: 97.5 to 99.4%, amino acid difference: 32 to 141 amino acids) across the genome (Supplementary Table 2). The sequences generated in this study are available from NCBI Genbank (accession numbers OM645180 to OM645191 and OP7753746 to OP753816).

**Table 1:** Cencurut virus (CENV)-positive *Suncus murinus* based RT-qPCR on tissues, sampling sites and habitat type in Singapore.

**District**	**Location**	**Habitat type**	**No. of CENV-positive/No. of shrew**			**Animal**
			**Lung**	**Spleen**	**Kidney**	
North	Central Water Catchment	Urban	2/3	3/3	3/3	**3/3**
North-East	Punggol	Urban	0/1	0/1	0/1	**0/1**
Central	Bukit Merah	Urban	4/9	5/9	7/9	**8/9**
	Bukit Timah	Urban	5/5	5/5	5/5	**5/5**
	Cantonment	Urban	0/2	0/2	2/2	**2/2**
	Marine Parade	Urban	1/2	1/2	2/2	**2/2**
	Outram	Urban	1/1	0/1	1/1	**1/1**
	Potong Pasir	Urban	1/1	1/1	1/1	**1/1**
	Rochor	Urban	1/1	1/1	1/1	**1/1**
	Bukit Timah	Forest⁎	2/3	2/3	3/3	**3/3**
	Cantonment	Forest	2/2	2/2	2/2	**2/2**
	Marine Parade	Forest	2/2	2/2	2/2	**2/2**
West	Clementi	Forest	3/5	3/5	4/5	**4/5**
**Total**			**24/37**	**25/37**	**33/37**	**34/37**

For full CENV RT-qPCR results, refer to Supplementary Table 1.

For full CENV RT-qPCR results, refer to Supplementary Table 1.

⁎ Forest = Young Secondary Forest/park.

**Fig. 3:**
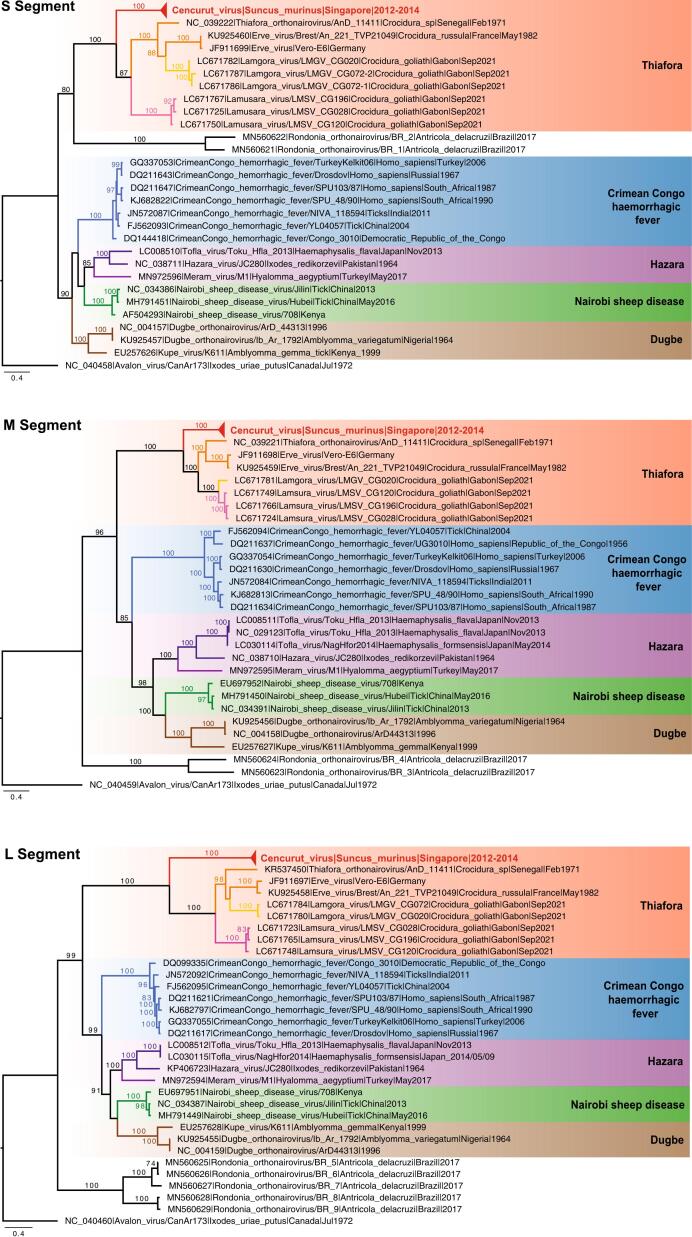
Maximum likelihood gene phylogenies of the S, M, and L nucleotide sequences of orthonairoviruses. Red text indicates novel Cencurut virus (CENV) sequences generated from this study. Colored branches denote different genogroups. Bootstrap values above 70% are shown at the major nodes. The scale bar indicates the number of nucleotide substitutions per site. (For interpretation of the references to colour in this figure legend, the reader is referred to the web version of this article.)

**Supplementary Fig. 3:**
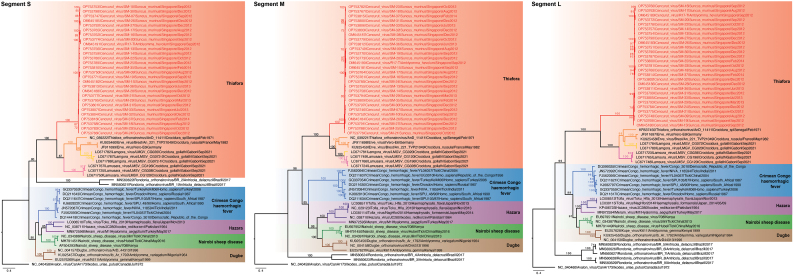
Maximum likelihood gene phylogenies of the S (n = 28), M (n = 28) and L (n = 27) nucleotide segments of orthonairoviruses. Red text indicates novel Cencurut virus (CENV) sequences generated from this study. Colored branches denote different genogroups. Bootstrap values above 70% are indicated at the major nodes. Scale bar indicates the number of nucleotide substitutions per site.

Maximum likelihood phylogenies of S, M, and L gene segments of orthonairoviruses from the Thiafora and Nairobi sheep disease genogroups show that the three gene segments of our 28 novel CENV strains (from 27 shrews and the tick) clustered together to form a strongly supported monophyletic clade (bootstrap = 100%, Fig. 3). CENV strains form a sister group to ERVEV,Lamgora virus (LMGV), Lamusara virus (LMSV), and Thiafora virus (TFAV) and are collectively nested within the well-supported Thiafora genogroup clade (bootstrap = 100%, Fig. S3). CENV in Singapore shares very low genetic similarity to these four viruses ranging from 49.0 to 58.2% nucleotide and 41.7.8 to 61.1% amino acid similarity only (Supplementary Table 3).

We also observed differences between the cleavage site in the S and M gene segments of Singapore CENV with other related orthonairoviruses (Fig. S4). Firstly, the caspase-3 cleavage motif (TVLD) located in the S gene segment of CENV is unique from that of ERVEV (DVLD), LMGV (NILE), LMSV (SIMD), and TFAV (DILD) (Figure 4A). This motif has been suggested to impact host cell apoptosis and viral replication in CCHFV and Hazara virus (HAZV) [33,34]. Secondly, the SKI-1 protease cleavage site in the M gene segment, responsible for cleaving the glycoprotein into the mature Gn and Gc glycoproteins, differs between the orthonairoviruses, with unique combinations of ([R][R/K/S][[L/P/I][L/K/N/S/M]) residues. Variation of these amino acid residues may be related to adaptation to various vector hosts [3]. Specifically, CENV had residues RKLM, whereas related ERVEV, LMGV, LMSV, and TFAV displayed RKLL, RRLL, and RQLL motifs (Fig. S4B, Fig. S4C).

## 4 Discussion

There are currently four shrew-borne orthonairoviruses classified under the Thiafora genogroup – Erve virus (ERVEV), Lamgora (LMGV), Lamusara virus (LMSV), and Thiafora virus (TFAV). Here, we detected and phylogenetically characterized a novel shrew-borne *Orthonairovirus* (Cencurut virus, CENV) in Asian house shrews (*Suncus murinus*) from Singapore. The newly identified CENV formed a monophyletic group within the Thiafora genogroup, reflecting clade-specific host and vector associations that extend across the genus *Orthonairovirus* [3]. However, CENV has only moderate genetic similarity to other Thiafora genogroup orthonairoviruses across all three gene segments and possesses unique amino acid combinations at the caspase-3 and SKI-1 protease cleavage sites.

We detected significantly higher viral loads in the kidneys of *S. murinus* than in the lung and spleen. However, tissue tropism cannot be conclusively determined as other tissues were not collected during this study. Despite high viral loads detected in the shrews, the animals appeared active, suggesting that this virus is endemic among Asian house shrews in Singapore and that shrews may act as a natural host in contributing to the persistence and genetic diversity of orthonairoviruses. Similarly, bicolored white-toothed shrews (*Crocidura leucodon*) from Austria have been shown to have a high detection rate (66.7%) of Borna disease virus-1 (BoDV-1) [35] while not exhibiting clinical infection despite high viral loads in various tissues, leading to speculation that infection persists throughout *C. leucodon*'s lifespan and demonstrating the potential as a BoDV-1 reservoir. There is a paucity of data on the persistence of orthonairoviruses in small mammals; however, ticks are persistently infected with CCHFV [36,37]. Within the order Bunyavirales (which includes the genus *Orthonairovirus*), small mammals are persistently infected with hantaviruses [38].

Shrews are widely distributed globally and are known carriers of several parasites, including helminths, bacteria, and viruses [39]. Asian house shrews can harbor various important human pathogens, including alphacoronavirus [40], hantavirus [41], mammarenavirus [42], hepatitis E virus [43], hepacivirus [44], and the plague bacterium (*Yersinia pestis*) [45], demonstrating their importance in the ecology of zoonotic diseases in the region. These mouse-sized insectivorous shrews are an invasive species probably originating in South Asia that are now widely distributed across Asia, the Middle East, and Eastern Africa [46]. They are synanthropic, exist in human-modified habitats with high human-contact interfaces, and are commonly encountered. Asian house shrews outnumber common pest *Rattus* species in Indonesian markets, where they have adapted to thrive on human foods [47]. In Singapore, Asian house shrews are common in urban areas, parks, and young secondary forests and are primarily active at night.

Orthonairoviruses are primarily arthropod-borne viruses, mainly detected in the ectoparasites of birds and mammals [1]. The role of ticks in emerging zoonoses in Southeast Asia is poorly understood, and the identification of these vectors is hampered by a paucity of taxonomic research and difficulty in assigning immature forms [48]. In this study, an *Amblyomma helvolum* specimen collected from an infected *S. murinus* was found to harbor a high CENV load, suggesting the consumption of a blood meal from its host and its potential as a vector. *Amblyomma helvolum* is often characterized as a reptile specialist and is commonly collected from lizards and snakes [48]. This is the only record of *A. helvolum* from a shrew; however, recent studies have recorded *A. helvolum* feeding on a broad range of mammals, including pigs, rodents, ruminants, and squirrels [[49], [50], [51]]. To incriminate this species as a vector, one would need to establish their competence (the ability of a species to transmit a parasite) and capacity (the combined factors that determine the host-vector encounters in natural environments) [52]. As the vectors of the shrew infecting ERVEV, LMGV, LMSV, and TFAV are currently unknown, the detection of CENV in *A. helvolum* suggests that viruses of the Thiafora genogroup may potentially be transmitted by ticks or other ectoparasites, like most orthonairoviruses.

The zoonotic potential of orthonairoviruses remains understudied globally. ERVEV seropositivity has been detected in humans, rodents, red deer, and herring gulls across several countries in Western Europe, and the virus has been implicated in causing headaches in humans [19,20,53]. In addition, serologic evidence for CCHFV infection in humans suggests an extensive geographical distribution ranging from Africa through Europe, the Middle East, and Asia [54]. However, there are few reports of *Orthonairovirus* species from Southeast Asia, likely due to a lack of sampling. Therefore, it is of public health importance to continue surveillance of orthonairvoriuses in highly prevalent synanthropic species such as the Asian house shrew. In particular, serosurveys are required to determine if immunological responses against CENV have been activated within humans to further our understanding of *Orthonairovirus* zoonotic risk.

The following are the supplementary data related to this article.

Supplementary Fig. 4 Alignment of the a) caspase-3 cleavage sites and SKI-1 cleavage site for b) Gn glycoprotein and c) Gc glycoprotein of Cencurut virus (CENV) and related Thiafora, Erve and Lamusara viruses. Supplementary Fig. 4

Supplementary Table 1 Amplification cycle threshold (ct) values and Cencurut virus (CENV) copies per mg of tissues or tick calculated from RT-qPCR standard curve. Supplementary Table 1

Supplementary Table 2 Pairwise comparisons of Cencurut virus genome (n = 28) in **a)** nucleotide and amino acid sequence similarity (%) and **b)** nucleotide and amino acid sequence differences. Supplementary Table 2

Supplementary Table 3 Pairwise comparisons of nucleotide and amino acid sequences of the **a)** L segment (RNA dependent RNA Polymerase), **b)** M segment (Glycoprotein) and **c)** S segment (Nucleocapsid) of representative Cencurut virus (CENV) with Thiafora virus (TFAV; KR537450, NC_039221, NC_039222), Erve virus (ERVEV; KU925458, KU925459, KU925460) and Lamusara virus (LMSV; LC671765, LC671766, LC671767). Supplementary Table 3

Supplementary materials and methods Supplementary material

## Ethical statement

Small mammal research was approved by the National University of Singapore Institutional Animal Care and Use Committee protocol B01/12, National Parks permit NP/RP12–004, and Agri-Food and Veterinary Authority of Singapore permit AV16.01.004.0004.

## CRediT authorship contribution statement

**Dolyce H.W. Low:** Conceptualization, Methodology, Formal analysis, Investigation, Data curation, Writing – original draft, Visualization. **Lena Ch'ng:** Validation, Investigation. **Yvonne C.F. Su:** Formal analysis, Writing – original draft, Writing – review & editing, Visualization. **Martin Linster:** Methodology. **Rong Zhang:** Formal analysis, Data curation. **Zhuang Yan:** Investigation. **Mackenzie L. Kwak:** Investigation. **Sophie A. Borthwick:** Investigation. **Alan T. Hitch:** Methodology, Formal analysis. **Gavin J.D. Smith:** Conceptualization, Writing – review & editing, Supervision. **Ian H. Mendenhall:** Conceptualization, Methodology, Writing – original draft, Writing – review & editing, Supervision.

## Declaration of Competing Interest

The authors declare the following financial interests/personal relationships which may be considered as potential competing interests: Gavin James Smith reports financial support and administrative support were provided by Duke-NUS Medical School Program in Emerging Infectious Diseases.

## Data Availability

All data is publicly available, links provided in manuscript
