# Supplementary material for: Cencurut virus: A novel Orthonairovirus from Asian house shrews (Suncus murinus) in Singapore
Source: One Health. 2023 Mar 29;16:100529. doi: 10.1016/j.onehlt.2023.100529 (PMC10288052; doi:10.1016/j.onehlt.2023.100529)
Supplement: Supplementary Fig. 4 — Alignment of the a) caspase-3 cleavage sites and SKI-1 cleavage site for b) Gn glycoprotein and c) Gc glycoprotein of Cencurut virus (CENV) and related Thiafora, Erve and Lamusara viruses. [file mmc4.pdf]

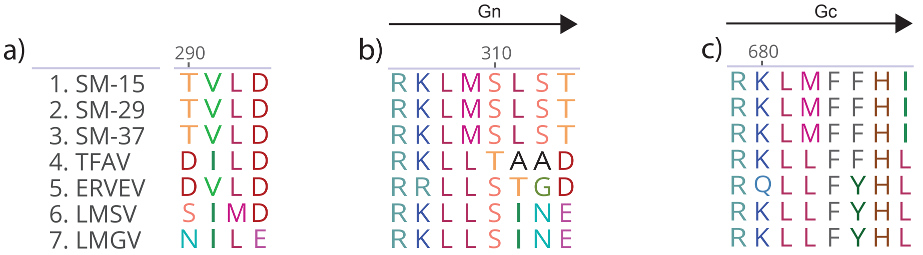
a)
290
1. SM-15 TVLD
2. SM-29 TVLD
3. SM-37 TVLD
4. TFAV DILD
5. ERVEV DVLD
6. LMSV SIMD
7. LMGV NILE
b)
Gn
310
RKLMSLST
RKLMSLST
RKLMSLST
RKLLTAAD
RRLLSTGD
RKLLSINE
RKLLSINE
c)
Gc
680
RKLMFFHI
RKLMFFHI
RKLMFFHI
RKLLFFHL
RQLLFYHL
RKLLFYHL
RKLLFYHL
