# Supplementary material for: Cencurut virus: A novel Orthonairovirus from Asian house shrews (Suncus murinus) in Singapore
Source: One Health. 2023 Mar 29;16:100529. doi: 10.1016/j.onehlt.2023.100529 (PMC10288052; doi:10.1016/j.onehlt.2023.100529)
Supplement: Supplementary Table 1 — Amplification cycle threshold (ct) values and Cencurut virus (CENV) copies per mg of tissues or tick calculated from RT-qPCR standard curve. [file mmc5.docx]

**Supplementary Table 1.** Amplification cycle threshold (ct) values and Cencurut virus (CENV) copies per mg of tissues or tick calculated from RT-qPCR standard curve.

| **Sample ID** | **Sampling site** | **Date of collection** | **Lung** | | **Spleen** | | **Kidney** | |
| --- | --- | --- | --- | --- | --- | --- | --- | --- |
|  |  |  | **ct value†** | **CENV copies per mg lung** | **ct value†** | **CENV copies per mg spleen** | **ct value†** | **CENV copies per mg kidney** |
| **SM-01** | Cantonment | 2012-06-15 | ND | ND | ND | ND | 34.03 | 8,248.20 |
| **SM-02** | Clementi | 2012-04-13 | ND | ND | ND | ND | ND | ND |
| **SM-03** | Outram | 2012-06-29 | 34.55 | 3,096.96 | ND | ND | 33.75 | 4,735.53 |
| **SM-04** | Clementi | 2012-04-14 | ND | ND | ND | ND | 30.17 | 46,662.32 |
| **SM-05** | Cantonment | 2012-07-26 | ND | ND | ND | ND | 34.41 | 2,963.00 |
| **SM-06** | Punggol | 2012-07-26 | ND | ND | ND | ND | ND | ND |
| **SM-07** | Bukit Merah | 2012-09-14 | ND | ND | 23.87 | 2,494,976.47 | ND | ND |
| **SM-08** | Bukit Merah | 2012-09-14 | ND | ND | ND | ND | ND | ND |
| **SM-09** | Marine Parade | 2012-08-21 | ND | ND | ND | ND | 27.34 | 93,921.18 |
| **SM-10** | Bukit Merah | 2012-09-15 | ND | ND | ND | ND | 28.50 | 130,780.20 |
| **SM-11** | Bukit Merah | 2012-09-14 | 33.15 | 9,782.35 | ND | ND | 29.81 | 32,367.45 |
| **SM-12** | Bukit Merah | 2012-09-14 | ND | ND | 33.48 | 18,938.35 | 32.11 | 11,561.91 |
| **SM-13** | Bukit Merah | 2012-09-15 | 21.00 | 32,199,968.66 | 26.80 | 320,268.67 | 19.00 | 37,208,627.16 |
| **SM-14** | Marine Parade | 2012-08-22 | 22.73 | 17,320,814.46 | 27.14 | 561,887.77 | 14.12 | 777,973,402.19 |
| **SM-15** | Bukit Merah | 2012-09-15 | 18.78 | 70,671,094.38 | 19.62 | 35,377,414.89 | 14.26 | 954,164,300.88 |
| **SM-16** | Bukit Merah | 2012-09-15 | 19.03 | 111,312,832.11 | ND | ND | 14.19 | 1,213,838,114.18 |
| **SM-17** | Potong Pasir | 2012-08-17 | 29.15 | 124,770.47 | 34.19 | 3,957.88 | 26.30 | 246,671.22 |
| **SM-18** | Rochor | 2012-08-16 | 20.49 | 20,473,541.11 | 16.15 | 158,151,702.19 | 15.18 | 730,973,043.75 |
| **SM-19** | Cantonment | 2012-09-28 | 18.71 | 108,641,241.04 | 20.58 | 12,736,727.41 | 18.63 | 171,369,222.41 |
| **SM-20** | Cantonment | 2012-09-24 | 28.49 | 87,049.07 | 23.05 | 5,072,441.09 | 20.59 | 5,746,535.14 |
| **SM-21** | Clementi | 2012-10-15 | 19.75 | 31,957,732.98 | 16.39 | 259,421,647.00 | 17.85 | 57,690,225.70 |
| **SM-22** | Clementi | 2012-10-17 | 30.19 | 47,895.21 | 19.40 | 16,375,387.70 | 18.61 | 34,363,762.46 |
| **SM-23** | Clementi | 2012-10-19 | 34.17 | 7,539.01 | 30.37 | 15,733.96 | 24.38 | 1,154,344.68 |
| **SM-24** | Bukit Merah | 2012-12-04 | ND | ND | 32.19 | 7,409.63 | 28.39 | 122,768.92 |
| **SM-25** | Bukit Timah | 2012-12-11 | 23.93 | 1,425,406.30 | 24.04 | 810,986.84 | 15.64 | 146,700,232.24 |
| **SM-26** | Bukit Timah | 2012-12-11 | 21.02 | 16,965,011.59 | 20.03 | 17,129,177.53 | 12.81 | 1,601,509,311.37 |
| **SM-27** | Marine Parade | 2012-12-19 | 33.90 | 2,212.41 | 32.78 | 6,031.73 | 21.52 | 8,708,026.43 |
| **SM-28** | Bukit Timah | 2012-12-11 | 20.22 | 26,848,799.55 | 18.94 | 25,410,973.82 | 16.15 | 116,354,466.61 |
| **SM-29** | CWC | 2013-03-07 | 20.27 | 29,040,785.01 | 17.83 | 92,897,365.43 | 15.77 | 275,788,261.42 |
| **SM-30** | Bukit Timah | 2012-12-11 | 20.58 | 25,862,861.36 | 18.90 | 30,770,528.43 | 17.08 | 156,540,782.06 |
| **SM-31** | Marine Parade | 2012-12-21 | 22.01 | 11,093,864.67 | 18.95 | 32,975,829.71 | 13.58 | 692,534,311.62 |
| **SM-32** | Bukit Timah | 2012-12-14 | 28.20 | 64,552.88 | 27.14 | 428,809.09 | 26.01 | 404,279.70 |
| **SM-33** | CWC | 2013-07-18 | ND | ND | 28.66 | 78,352.67 | 28.51 | 68,879.22 |
| **SM-34** | Bukit Timah | 2014-02-25 | ND | ND | 33.14 | 4,520.55 | 24.82 | 3,686,108.95 |
| **SM-35** | Bukit Timah | 2014-02-26 | 21.51 | 7,398,109.83 | 17.67 | 58,563,029.79 | 15.01 | 311,677,285.65 |
| **SM-36** | CWC | 2013-06-27 | 20.13 | 20,889,116.06 | 20.49 | 9,858,886.37 | 17.78 | 21,187,138.29 |
| **SM-37** | Bukit Timah | 2014-02-25 | 30.79 | 19,322.40 | ND | ND | 28.63 | 57,545.28 |
| **Tick** | SM-20 | 2012-09-24 | 27.86 | 1,133,379.47 |  |  |  |  |
| †Limit of Quantification (LoQ) cut off at ct 34.59 (149.37 copies per reaction)  ND, not determined | | | | | | | | |
| CWC, Central Water Catchment | | | | | | | | |
