# Supplementary material for: Cencurut virus: A novel Orthonairovirus from Asian house shrews (Suncus murinus) in Singapore
Source: One Health. 2023 Mar 29;16:100529. doi: 10.1016/j.onehlt.2023.100529 (PMC10288052; doi:10.1016/j.onehlt.2023.100529)
Supplement: Supplementary Table 2 — Pairwise comparisons of Cencurut virus genome (n = 28) in a) nucleotide and amino acid sequence similarity (%) and b) nucleotide and amino acid sequence differences. [file mmc6.pdf]

a)

Nucleotide similarity (%) (upper triangle); Amino acid similarity (%) (lower triangle)

Sample ID	SM-07*	SM-09*	SM-10	SM-13	SM-14	SM-15	SM-16	SM-17	SM-18	SM-19	SM-20	SM-21	SM-22	SM-23	SM-24	SM-25	SM-26	SM-27	SM-28	SM-29	SM-30	SM-31	SM-32	SM-33	SM-35	SM-36	SM-37	Tick
**SM-07***		92.7	95.7	95.0	94.0	93.8	93.9	93.9	94.6	94.2	94.5	94.5	94.0	94.6	94.6	94.3	94.6	94.1	94.5	94.3	95.1	93.9	94.9	94.5	93.7	93.6	94.2	93.5
**SM-09***	93.4		93.9	93.0	93.3	93.5	93.2	93.2	94.0	95.3	94.3	93.9	93.9	93.9	94.1	93.6	93.5	93.5	93.6	93.9	93.8	93.4	94.7	95.5	93.1	93.3	93.5	93.3
**SM-10**	96.1	95.0		97.8	96.5	96.4	95.6	95.6	96.3	96.3	96.6	96.3	96.0	96.3	96.4	96.2	96.7	96.3	96.0	96.1	96.9	95.7	96.1	95.9	95.4	95.4	95.8	95.3
**SM-13**	96.2	94.3	98.6		96.5	96.4	95.3	95.3	96.0	96.1	96.6	95.8	95.7	96.0	96.0	95.7	95.9	95.5	95.8	95.7	95.5	95.5	95.6	95.5	95.2	95.2	95.4	95.1
**SM-14**	95.8	94.7	98.1	98.2		98.3	95.4	95.4	96.1	96.1	96.4	95.9	95.8	96.0	96.1	95.7	96.0	95.6	96.0	95.7	95.6	95.5	95.9	95.6	95.0	95.1	95.5	95.1
**SM-15**	95.8	94.6	98.1	98.3	99.2		95.4	95.4	95.9	96.7	96.9	95.8	95.7	95.9	95.9	95.6	95.8	95.5	95.9	95.6	95.5	95.4	95.6	95.4	95.0	95.0	95.3	95.6
**SM-16**	96.2	94.7	97.9	98.0	98.1	98.3		100.0	97.8	97.0	97.0	95.7	95.5	95.8	96.0	95.7	95.7	95.5	95.6	95.5	95.8	95.5	95.5	95.3	95.0	95.1	95.4	95.4
**SM-17**	96.2	94.7	97.9	98.0	98.1	98.3	100.0		97.8	97.0	97.0	95.7	95.5	95.8	96.0	95.7	95.7	95.5	95.6	95.5	95.8	95.5	95.5	95.3	95.0	95.1	95.4	95.4
**SM-18**	96.3	95.0	98.3	98.2	98.4	98.4	98.9	98.9		97.3	97.2	96.6	96.4	97.9	97.3	96.4	96.4	96.4	96.7	96.4	96.4	96.8	96.9	96.8	95.7	95.9	97.0	95.6
**SM-19**	95.9	95.5	98.2	98.2	98.3	98.5	98.9	98.9	98.9		97.9	97.3	96.5	96.9	97.0	96.6	96.4	96.5	96.5	96.4	96.2	96.2	96.8	96.7	95.8	95.8	96.2	96.5
**SM-20**	96.3	95.1	98.2	98.2	98.3	98.5	98.9	98.9	98.8	99.1		96.7	96.8	97.1	97.1	96.6	96.6	96.5	96.6	96.5	96.4	96.4	96.6	96.4	95.9	95.9	96.3	97.7
**SM-21**	96.3	94.9	98.0	97.9	98.1	98.2	98.3	98.3	98.9	98.9	98.4		96.4	96.8	96.9	96.9	96.3	96.6	96.3	96.2	96.1	96.0	96.5	96.2	95.4	95.5	95.9	95.4
**SM-22**	96.1	94.8	98.1	97.9	98.1	98.1	98.1	98.1	98.7	98.5	98.6	98.4		98.5	96.6	96.4	96.1	96.3	96.2	96.0	95.8	95.9	96.2	96.1	95.3	95.6	95.9	95.3
**SM-23**	96.2	94.8	98.0	97.9	98.1	98.1	98.3	98.3	99.3	98.7	98.7	98.6	99.4		97.5	96.7	96.3	96.6	96.6	96.3	96.1	96.7	97.0	97.0	95.7	95.9	96.9	95.5
**SM-24**	96.4	95.0	98.1	98.0	98.2	98.3	98.5	98.5	99.1	98.8	98.7	98.8	98.7	99.1		96.7	96.3	96.6	96.5	96.3	96.2	96.4	96.9	96.8	95.6	95.8	96.6	95.7
**SM-25**	96.3	94.9	98.0	97.9	98.2	98.2	98.3	98.3	98.7	98.7	98.5	98.6	98.7	98.7	98.7		96.3	96.5	96.1	96.0	96.0	95.8	96.3	96.0	95.4	95.5	95.8	95.3
**SM-26**	96.2	94.7	98.2	98.0	98.2	98.2	98.4	98.4	98.7	98.4	98.4	98.3	98.4	98.5	98.5	98.4		97.2	96.2	96.0	96.4	95.9	96.0	95.7	95.5	95.5	95.8	95.4
**SM-27**	96.1	94.8	98.1	97.8	98.1	98.1	98.2	98.2	98.9	98.5	98.5	98.5	98.6	98.7	98.8	98.6	98.9		95.9	95.9	96.3	95.7	96.2	95.9	95.3	95.3	95.6	95.2
**SM-28**	96.3	94.6	97.9	97.9	98.2	98.2	98.1	98.1	98.6	98.5	98.4	98.3	98.4	98.4	98.4	98.3	98.4	98.3		96.0	95.7	96.0	96.2	96.0	95.4	95.7	96.1	95.4
**SM-29**	96.1	95.1	98.0	97.9	98.1	98.1	98.1	98.1	98.6	98.4	98.4	98.3	98.4	98.5	98.5	98.3	98.4	98.4	98.2		95.9	95.9	96.0	95.9	95.5	95.5	95.9	95.4
**SM-30**	96.4	94.9	98.4	97.8	98.0	98.1	98.3	98.3	98.6	98.5	98.4	98.3	98.3	98.3	98.4	98.4	98.5	98.5	98.2	98.2		95.7	95.9	95.6	95.3	95.2	95.7	95.3
**SM-31**	95.8	94.3	97.7	97.7	97.8	97.9	98.0	98.0	98.8	98.4	98.3	98.1	98.2	98.5	98.3	98.0	98.2	98.2	98.1	98.1	98.1		96.2	96.5	95.2	95.5	96.2	95.3
**SM-32**	96.4	95.1	97.9	97.8	98.1	98.1	98.3	98.3	99.0	98.7	98.5	98.5	98.5	98.9	98.8	98.6	98.3	98.5	98.3	98.3	98.2	98.4		98.1	95.4	95.6	96.2	95.3
**SM-33**	96.4	95.1	97.9	97.9	98.0	98.1	98.2	98.2	99.0	98.7	98.5	98.5	98.5	98.9	98.7	98.5	98.3	98.5	98.3	98.2	98.3	98.6	99.4		95.2	95.4	96.1	95.1
**SM-35**	96.1	94.6	97.6	97.7	97.6	97.7	97.9	97.9	98.3	98.1	98.1	97.8	97.9	98.0	98.0	98.0	97.9	97.9	98.0	98.0	97.8	97.7	98.0	98.0		98.3	96.5	96.0
**SM-36**	96.1	94.5	97.5	97.5	97.5	97.6	97.8	97.8	98.3	98.0	98.0	97.8	97.9	98.0	98.0	97.8	97.9	97.9	98.0	97.9	97.7	97.7	98.0	98.0	99.3		96.6	95.9
**SM-37**	96.3	94.5	97.7	97.7	97.9	97.9	98.1	98.1	98.8	98.3	98.2	98.1	98.2	98.5	98.4	98.2	98.2	98.2	98.2	98.2	98.0	98.2	98.5	98.5	98.3	98.3		98.6
**Tick**	95.9	94.5	97.6	97.6	97.8	98.0	98.1	98.1	98.3	98.5	98.8	97.9	97.9	98.0	98.1	98.0	98.0	98.0	98.0	97.9	97.9	97.8	98.0	98.0	98.1	98.0	99.4	

* partial genomes

b)

Nucleotide differences (upper triangle); Amino acid differences (lower triangle)

Sample ID	SM-07*	SM-09*	SM-10	SM-13	SM-14	SM-15	SM-16	SM-17	SM-18	SM-19	SM-20	SM-21	SM-22	SM-23	SM-24	SM-25	SM-26	SM-27	SM-28	SM-29	SM-30	SM-31	SM-32	SM-33	SM-35	SM-36	SM-37	Tick
**SM-07***		345	289	331	398	412	405	405	360	385	365	365	397	362	361	378	361	390	366	377	326	402	339	365	415	427	386	432
**SM-09***	98		627	715	695	667	695	695	627	492	588	632	632	633	607	661	675	670	664	628	644	679	552	471	713	691	666	685
**SM-10**	83	163		384	602	617	763	763	647	637	586	642	684	646	630	659	580	633	686	666	530	733	673	708	789	793	731	806
**SM-13**	82	191	79		596	611	791	791	691	669	590	720	731	687	678	731	694	760	708	733	769	763	746	767	823	815	776	831
**SM-14**	90	176	107	101		286	785	785	681	666	617	696	716	681	669	725	691	742	689	731	753	764	707	751	846	835	770	839
**SM-15**	90	180	105	97	48		776	776	705	571	533	717	734	705	698	749	715	766	705	753	771	788	742	777	850	849	793	755
**SM-16**	83	176	116	113	107	95		1	387	513	513	736	771	711	687	737	728	761	756	771	717	772	759	798	851	842	775	777
**SM-17**	83	176	116	113	107	95	0		387	513	513	736	771	711	687	737	728	761	756	771	717	772	759	798	851	842	775	777
**SM-18**	80	166	97	101	89	89	65	65		473	494	595	619	370	475	615	630	614	578	628	625	555	538	560	735	703	526	751
**SM-19**	89	152	102	104	97	84	63	63	60		360	461	594	539	521	584	622	606	603	626	658	644	554	571	724	715	648	599
**SM-20**	80	165	100	102	98	83	64	64	66	53		561	544	501	506	583	582	594	582	601	614	609	591	624	697	703	633	400
**SM-21**	80	171	114	120	108	103	95	95	65	65	88		616	555	523	533	630	585	635	650	663	678	593	645	783	775	695	784
**SM-22**	85	172	108	120	107	108	105	105	72	85	81	88		255	576	616	664	632	649	678	706	693	641	663	792	755	705	793
**SM-23**	82	174	112	117	105	105	95	95	37	76	74	77	35		430	568	629	587	589	625	663	564	508	510	742	711	535	760
**SM-24**	78	168	106	112	100	97	84	84	52	70	73	67	72	53		556	632	577	597	626	646	616	527	556	744	725	581	733
**SM-25**	80	170	113	120	102	102	97	97	71	76	83	79	76	73	72		636	596	669	679	673	711	637	676	775	769	710	794
**SM-26**	82	177	100	114	104	103	92	92	71	88	88	95	89	86	83	91		472	654	689	616	707	683	729	772	767	722	789
**SM-27**	84	173	108	122	109	108	102	102	65	84	87	84	79	73	68	80	61		699	703	635	725	651	704	804	803	743	809
**SM-28**	80	181	119	119	104	103	105	105	77	87	88	95	91	89	89	97	91	98		684	726	684	648	677	780	734	672	789
**SM-29**	84	164	114	121	110	108	105	105	77	91	93	96	93	85	87	97	92	93	102		695	693	677	705	757	763	691	785
**SM-30**	77	169	90	126	113	110	98	98	77	84	88	97	96	96	92	93	83	87	103	101		736	705	750	805	811	726	801
**SM-31**	92	191	129	130	123	121	111	111	69	91	95	106	103	84	94	111	104	101	108	109	110		643	602	808	766	642	797
**SM-32**	79	165	116	122	105	108	96	96	54	76	84	84	85	61	70	84	94	84	96	97	100	90		330	787	751	642	809
**SM-33**	78	165	118	120	112	110	104	104	59	75	85	87	86	63	72	86	96	86	95	100	99	77	34		819	779	659	832
**SM-35**	84	181	135	132	134	128	118	118	95	105	108	123	119	113	113	114	117	117	114	115	123	131	112	115		286	602	679
**SM-36**	85	185	143	139	141	135	124	124	98	113	111	127	120	115	115	122	118	119	112	117	128	132	114	115	39		583	695
**SM-37**	81	184	132	133	120	120	110	110	68	94	101	107	104	84	92	104	100	101	102	104	115	100	84	87	95	98		237
**Tick**	88	185	137	138	127	112	106	106	99	83	69	119	118	115	108	111	113	114	114	119	120	126	115	115	106	111	32	

* partial genomes
