# Supplementary material for: Cencurut virus: A novel Orthonairovirus from Asian house shrews (Suncus murinus) in Singapore
Source: One Health. 2023 Mar 29;16:100529. doi: 10.1016/j.onehlt.2023.100529 (PMC10288052; doi:10.1016/j.onehlt.2023.100529)
Supplement: Supplementary Table 3 — Pairwise comparisons of nucleotide and amino acid sequences of the a) L segment (RNA dependent RNA Polymerase), b) M segment (Glycoprotein) and c) S segment (Nucleocapsid) of representative Cencurut virus (CENV) with Thiafora virus (TFAV; KR537450, NC_039221, NC_039222), Erve virus (ERVEV; KU925458, KU925459, KU925460) and Lamusara virus (LMSV; LC671765, LC671766, LC671767). [file mmc7.docx]

**Supplementary Table 5.** Pairwise comparisons of nucleotide and amino acid sequences of the A) S segment, B) M segment and C) L segment of representative Cencurut virus (CENV) with Thiafora virus (TFAV; NC_039222, NC_039221, KR537450), Erve virus (ERVEV; KU925460, KU925459, KU925458), Lamusara virus (LMSV; LC671767, LC671766, LC671765) and Lamgora virus (LMGV; LC671782, LC671781, LC671780).

| **a)** |  | **Sample ID** | **SM-15** | **SM-29** | **SM-37** | **TFAV** | **ERVEV** | **LMSV** | **LMGV** |
| --- | --- | --- | --- | --- | --- | --- | --- | --- | --- |
|  |  |  | **S segment nucleotide similarity (%)** | | | | | | |
|  |  | **SM-15** |  | 94.8 | 94.8 | 49.1 | 50.0 | 49.4 | 52.2 |
|  |  | **SM-29** | 96.9 |  | 96.2 | 49.0 | 50.5 | 49.3 | 51.4 |
|  |  | **SM-37** | 97.6 | 99.0 |  | 49.1 | 49.9 | 49.2 | 52.4 |
|  |  | **TFAV** | 41.7 | 41.8 | 41.8 |  | 60.4 | 49.5 | 61.5 |
|  |  | **ERVEV** | 44.5 | 44.1 | 44.3 | 57.4 |  | 53.1 | 61.7 |
|  |  | **LMSV** | 45.5 | 45.3 | 45.6 | 43.3 | 46.2 |  | 49.8 |
|  |  | **LMGV** | 44.2 | 44.1 | 44.3 | 62.5 | 61.9 | 44.3 |  |
|  |  |  | **S segment amino acid similarity (%)** | | | | | | |
|  |  |  |  |  |  |  |  |  |  |
| **b)** |  | **Sample ID** | **SM-15** | **SM-29** | **SM-37** | **TFAV** | **ERVEV** | **LMSV** | **LMGV** |
|  |  |  | **M segment nucleotide similarity (%)** | | | | | | |
|  |  | **SM-15** |  | 95.3 | 96.1 | 57.4 | 58.0 | 57.0 | 57.4 |
|  |  | **SM-29** | 98.2 |  | 97.4 | 57.7 | 58.1 | 56.7 | 57.3 |
|  |  | **SM-37** | 98.2 | 98.8 |  | 57.8 | 58.2 | 56.8 | 57 |
|  |  | **TFAV** | 59.6 | 59.8 | 59.6 |  | 68.7 | 65.6 | 65 |
|  |  | **ERVEV** | 60.7 | 61.0 | 61.1 | 71.0 |  | 64.0 | 64.2 |
|  |  | **LMSV** | 60.0 | 60.1 | 59.7 | 68.7 | 66.7 |  | 81.5 |
|  |  | **LMGV** | 59.3 | 59.5 | 59.1 | 66.9 | 66.9 | 90 |  |
|  |  |  | **M segment amino acid similarity (%)** | | | | | | |
|  |  |  |  |  |  |  |  |  |  |
| **c)** |  | **Sample ID** | **SM-15** | **SM-29** | **SM-37** | **TFAV** | **ERVEV** | **LMSV** | **LMGV** |
|  |  |  | **L segment nucleotide similarity (%)** | | | | | | |
|  |  | **SM-15** |  | 96.1 | 96.1 | 57.1 | 58.0 | 57.6 | 57.8 |
|  |  | **SM-29** | 98.6 |  | 96.7 | 57.4 | 58.1 | 57.4 | 58 |
|  |  | **SM-37** | 98.8 | 98.9 |  | 57.0 | 57.8 | 57.4 | 57.8 |
|  |  | **TFAV** | 54.6 | 54.8 | 54.6 |  | 68.1 | 67.0 | 69.9 |
|  |  | **ERVEV** | 55.1 | 55.2 | 55.0 | 72.0 |  | 67.3 | 69.1 |
|  |  | **LMSV** | 53.8 | 53.9 | 53.8 | 69.8 | 68.9 |  | 68.6 |
|  |  | **LMGV** | 54.0 | 54.0 | 54.1 | 74.6 | 72.8 | 70.2 |  |
|  |  |  | **L ge segment ne amino acid similarity (%)** | | | | | | |
