# Supplementary material for: Cencurut virus: A novel Orthonairovirus from Asian house shrews (Suncus murinus) in Singapore
Source: One Health. 2023 Mar 29;16:100529. doi: 10.1016/j.onehlt.2023.100529 (PMC10288052; doi:10.1016/j.onehlt.2023.100529)
Supplement: Supplementary materials and methods [file mmc8.docx]

Supplementary methods

Library preparation: A cDNA template was synthesized from 50ng of tick-derived RNA by firstly incubating the RNA with 50µM Random Primer 6 (New England BioLabs, Ipswich, USA) at 95°C for 5 mins and cooling to 20°C with a ramp rate of 0.1-0.2°C per second. First strand cDNA was subsequently synthesized from the previous step in a 50µl reaction consisting of 20µl RNA-Random Primer 6 mix, 25µl ProtoScript II Reaction Mix and 5µl ProtoScript II Enzyme Mix (ProtoScript® II First Strand cDNA Synthesis Kit, New England BioLabs, Ipswich, USA) at 25°C for 5 mins, 42°C for 1 hr, 80°C for 5 mins and a hold at 4°C. Second strand cDNA was synthesized from the first strand in a 80µl reaction consisting of 18µl Nuclease-free water, 8µl Second Strand Synthesis Reaction Buffer, 4µl Second Strand Synthesis Enzyme Mix (NEBNext® Ultra II Non-Directional RNA Second Strand Synthesis Module, New England BioLabs, Ipswich, USA) at 16°C for 1 hr. The tick cDNA template was purified with 1.2x DNA Purification beads with two 80% ethanol washes and elution with 25µl Nuclease-free water, with a subsequent dilution to 1ng/µl. Fragmentation, end repair and dA-trailing was carried out in a 50µl reaction consisting of 25µl DNA, 10µl Nuclease-free water, 5µl 10x Fragmentation Buffer and 10µl 5X Fragmentation Enzyme at the following conditions of 32°C for 22 mins, 65°C for 30 mins and a hold at 4°C. Adapters were ligated to the cDNA in a 50µl reaction containing 2.5µl Twist Universal Adapters, 17.5µl Nuclease-free water, 20µl DNA Ligation Buffer and 10µl DNA Ligation Mix at 20°C for 15 mins, and purified with 0.8x DNA Purification beads with three 80% ethanol washes and elution with 15µl Nuclease-free water. 10µl of Universal dual indexes were ligated to the adapted cDNA by amplification with 25µl KAPA HiFi HotStart ReadyMix (Roche Holding AG, Basel, Switzerland) at the following conditions of initialization at 98°C for 45 secs, 12 cycles of denaturation at 98°C for 15 secs, annealing at 60°C for 30 secs, extension at 72°C for 30 secs, a final extension at 72°C for 1 min, and holding at 4°C. The indexed cDNA library was purified with 1x DNA Purification beads with two 80% ethanol washes and elution with 20µl Nuclease-free water. Purified indexed cDNA libraries derived from ticks were quantified with a Qubit dsDNA BR Assay kit (Invitrogen, Waltham, USA) and library size profile visualized with a Bioanalyzer High Sensitivity DNA Assay kit (Agilent Technologies, Santa Clara, USA).

Enrichment: 500ng of the tick cDNA library was pooled in an 8-plex reaction (total 1,500ng), dried with a low heat vacuum concentrator, resuspended with 5µl human Blocker solution and 7µl Universal Blockers, and subsequently heated at 95°C for 5 mins. Simultaneously, a 28µl probe solution containing 20µl Hybridization Mix, 4µl Comprehensive Viral Research Panel and 4µl custom probe panel was prepared, heated at 95°C for 2 mins and immediately cooled on ice for 5 mins. Both cDNA library and probe solution were equilibrated to room temperature and combined, with the addition of 30µl Hybridization Enhancer on the top of the mixture and incubated at 70°C for 16 hrs. The hybridized reaction was added directly from the thermal cycler at 70°C to 200µl of Binding Buffer resuspended Streptavidin beads and incubated at room temperature, on a shaker for 30 mins. The enriched cDNA library bounded Streptavidin beads was washed once with 200µl of Wash Buffer 1, followed by three rounds of washing with 200µl of 48°C Wash Buffer 2, with 5 mins of incubation at 48°C between each wash, and resuspended in 45µl of Nuclease-free water. 22.5µl of the Streptavidin Binding Bead slurry was amplified in a 50µl reaction containing 2.5µl ILMN Amplification Primers and 25µl KAPA HiFi HotStart ReadyMix (Roche Holding AG, Basel, Switzerland) at the following conditions of initialization at 98°C for 45 secs, 19 cycles of denaturation at 98°C for 15 secs, annealing at 60°C for 30 secs, extension at 72°C for 30 secs, a final extension at 72°C for 1 min, and holding at 4°C. The amplified Streptavidin Binding Bead slurry was purified with 1.8x DNA Purification beads with two 80% ethanol washes and eluted with 30µl Nuclease-free water. The eluted enriched library was quantified with a Qubit dsDNA BR Assay kit (Invitrogen, Waltham, USA) and library size profile visualized with a Bioanalyzer High Sensitivity DNA Assay kit (Agilent Technologies, Santa Clara, USA).

Supplementary figures and tables

**Supplementary Figure 1**. *Amblyomma helvolum* nymph (A: dorsal view; B: ventral view).

**Supplementary Figure 2**. Hierarchal Bayesian model calculating the probability of a) sex, b) age and c) habitat type affecting the probability of *Suncus murinus* being tested positive for Cencurut virus (CENV) by RT-qPCR.

**Supplementary Figure 3**. Maximum likelihood gene phylogenies of the S (n=28), M (n=28) and L (n=27) nucleotide segments of orthonairoviruses. Red text indicates novel Cencurut virus (CENV) sequences generated from this study. Colored branches denote different genogroups. Bootstrap values above 70% are indicated at the major nodes. Scale bar indicates the number of nucleotide substitutions per site.

**Supplementary Figure 4.** Alignment of the **a)** caspase-3 cleavage sites and SKI-1 cleavage site for **b)** Gn glycoprotein and **c)** Gc glycoprotein of Cencurut virus (CENV) and related Thiafora, Erve and Lamusara viruses.

**Supplementary Table 1**. Amplification cycle threshold (ct) values and Cencurut virus (CENV) copies per mg of tissues or tick calculated from RT-qPCR standard curve.

**Supplementary Table 2**. Pairwise comparisons of Cencurut virus genome (n=28) in **a)** nucleotide and amino acid sequence similarity (%) and **b)** nucleotide and amino acid sequence differences.

**Supplementary Table 3.** Pairwise comparisons of nucleotide and amino acid sequences of the **a)** L segment (RNA dependent RNA Polymerase), **b)** M segment (Glycoprotein) and **c)** S segment (Nucleocapsid) of representative Cencurut virus (CENV) with Thiafora virus (TFAV; KR537450, NC_039221, NC_039222), Erve virus (ERVEV; KU925458, KU925459, KU925460) and Lamusara virus (LMSV; LC671765, LC671766, LC671767).
